# Gentamicin supplemented polyvinylidenfluoride mesh materials enhance tissue integration due to a transcriptionally reduced MMP-2 protein expression

**DOI:** 10.1186/1471-2482-12-1

**Published:** 2012-01-13

**Authors:** Marcel Binnebösel, Klaus T von Trotha, Christina Ricken, Christian D Klink, Karsten Junge, Joachim Conze, Marc Jansen, Ulf P Neumann, Petra Lynen Jansen

**Affiliations:** 1 Department of General, Visceral and Transplantation Surgery, RWTH Aachen University Hospital, Aachen, Germany; 2 Department of General, Visceral and Minimal Invasive Surgery, Helios Hospital Emil von Behring, Berlin, Germany

**Keywords:** mesh, gentamicin, PVDF, matrix metalloproteinase 2, wound healing

## Abstract

**Background:**

A beneficial effect of gentamicin supplemented mesh material on tissue integration is known. To further elucidate the interaction of collagen and MMP-2 in chronic foreign body reaction and to determine the significance of the MMP-2-specific regulatory element (RE-1) that is known to mediate 80% of the MMP-2 promoter activity, the spatial and temporal transcriptional regulation of the MMP-2 gene was analyzed at the cellular level.

**Methods:**

A PVDF mesh material was surface modified by plasma-induced graft polymerization of acrylic acid (PVDF+PAAc). Three different gentamicin concentrations were bound to the provided active sites of the grafted mesh surfaces (2, 5 and 8 μg/mg). 75 male transgenic MMP-2/LacZ mice harbouring the LacZ reporter gene under control of MMP-2 regulatory sequence -1241/+423, excluding the RE-1 were randomized to five groups. Bilateral of the abdominal midline one of the five different meshes was implanted subcutaneously in each animal. MMP-2 gene transcription (anti-ß-galactosidase staining) and MMP-2 protein expression (anti-MMP-2 staining) were analyzed semiquantitatively by immunohistochemistry 7, 21 and 90 days after mesh implantation. The collagen type I/III ratio was analyzed by cross polarization microscopy to determine the quality of mesh integration.

**Results:**

The perifilamentary ß-galactosidase expression as well as the collagen type I/III ratio increased up to the 90^th ^day for all mesh modifications, whereas no significant changes could be observed for MMP-2 protein expression between days 21 and 90. Both the 5 and 8 μg/mg gentamicin group showed significantly reduced levels of ß-galactosidase expression and MMP-2 positive stained cells when compared to the PVDF group on day 7, 21 and 90 respectively (5 μg/mg: p < 0.05 each; 8 μg/mg: p < 0.05 each). Though the type I/III collagen ratio increased over time for all mesh modifications significant differences to the PVDF mesh were only detected for the 8 μg/mg group at all 3 time points (p < 0.05 each).

**Conclusions:**

Our current data indicate that lack of RE-1 is correlated with increased mesh induced MMP-2-gene expression for coated as well as for non-coated mesh materials. Gentamicin coating reduced MMP-2 transcription and protein expression. For the 8 μg/mg group this effect is associated with an increased type I/III collagen ratio. These findings suggest that gentamicin is beneficial for tissue integration after mesh implantation, which possibly is mediated via RE-1.

## Background

Modern hernia surgery is no longer imaginable without the application of mesh prosthesis leading to millions of biomaterial implantations each year worldwide [1-3]. Implantation of alloplastic mesh material results in an inflammatory reaction to foreign bodies of a different nature that is surprisingly constant, characterized by a rapid accumulation of huge numbers of phagocytic cells, in particular blood monocytes and tissue-derived macrophages [4-6]. This type of inflammation is known as foreign body reaction (FBR), which is characterized by a transcriptionally induced overexpression of the matrix metalloproteinases 2 (MMP-2) [7-9].

MMP-2 (gelatinase A) plays an essential role in angiogenesis, inflammation, and fibrosis, and is necessary for a proper wound healing [10,11]. Advanced investigations by *Jansen *et al. revealed that mesh implantation mediates enhanced MMP-2 gene transcription with concomitantly up-regulated MMP-2 protein synthesis and enzymatic activity, thus resulting in a chronic inflammatory reaction [9]. Interestingly, a close correlation between MMP-2 expression and collagen formation and degradation is evident establishing a role for MMP-2 in critical events during wound repair [12]. Affecting MMP-2 by modification of either the polymer itself or by coating of the mesh material is suggested to be a potential approach to reduce the chronic inflammatory reaction to alloplastic mesh materials, and thereby to reduce long-term complications like mesh shrinkage, migration, adhesion and in particular chronic pain [13,14]. Furthermore, it is well known that the quality of perifilamentary scar formation which is characterized by the collagen type I/III ratio is of major impact to minimize the risk of complications or even to avoid the development of a recurrent hernia [15,16].

In a previously published study gentamicin coated polyvinylidenfluoride (PVDF) mesh material was detected to improve tissue integration due to an increased type I/III collagen ratio and a reduced MMP-2 protein expression [17]. However, our model analysing MMP-2 gene expression in transgenic mice revealed distinct MMP-2 promoter activation dependent on the course of time and on the concentration of gentamicin [17]. To further elucidate our proven beneficial effect of biomaterial supplementation on foreign body reaction and tissue integration an in-depth understanding of the gentamicin induced enhancement of MMP-2 is necessary. Therefore, gentamicin coated mesh materials with three different concentrations per unit of weight (μg/mg) were implanted in transgenic reporter mice harbouring a ß-galactosidase reporter gene (LacZ) driven by the regulatory sequences of the MMP-2 gene. To determine the significance of the response element-1 (RE-1) that extends from -1282/-1322, F8del mice were created that harbour MMP-2 regulatory sequence -1241/+423, thereby excluding the RE-1. The spatial and temporal transcriptional regulation of the MMP-2 gene was analyzed at the cellular level.

## Methods

### Animal studies

The experiments were officially approved by the local Animal Care and Use Review Committee (50.203.2-AC46, 38/02). All animals received humane care in accordance with the requirements of the German Tierschutzgesetz, §8 Abs. 1 and in accordance to the *Guide for the Care and Use of Laboratory Animals *published by the National Institute of Health.

A total of 75 male MMP-2/LacZ transgenic CD1-tg mice (mean body weight 28.2 ± 2.0 g) were randomly divided into five groups according to the type of mesh material used. All animals were kept under standardized conditions: temperature between 22°C and 24°C; relative humidity 50-60%; 12 h of light following 12 h of darkness. The animals had free access to food and water. Food was withdrawn 12 h before and after surgery. All operations were carried out under general anesthesia and aseptic and sterile surgical conditions.

### MMP-2/LacZ transgenic mice model

The mice strain used as animal model has been described recently [9,11,17,18]. In brief, mouse strain F8 harbour a β-galactosidase reporter gene (LacZ) under control of MMP-2 regulatory sequences -1686/+423 that extend to the middle of the second exon. Thereby cells with MMP-2 promoter activity can be detected by a monoclonal anti-β-galactosidase antibody. A part of this regulatory sequence is the response element-1 (RE-1) that extends from -1282/-1322. In previous data a crucial role for the enhancer element RE-1 was proven in injury-induced MMP-2 transcription of the skin (11). To determine the significance of the response element-1 (RE-1) in foreign body reaction and tissue integration in response to the surface modified biomaterials, the sequences -1282/-1322 were deleted and F8del mice were created that harbour MMP-2 regulatory sequence -1241/+423, thereby excluding the RE-1. Seventy five of those mice were used for the investigations.

### Mesh material

Overall five different mesh modifications with 0.5 × 0.5 cm in size were implanted: PVDF, a low-weight, large porous and elastic mesh made of polyvinylidenfluoride monofilaments (FEG Textiltechnik mbH, Aachen, Germany) was the basic prosthetic material for the construction of all mesh samples. Plasma-induced graft polymerization was used to modify the surface chemistry and morphology of the PVDF mesh samples [19]. Immediately after treatment, oxygen was introduced into the chamber to generate hydroperoxide as well as other functional groups on the sample surface (FEG Textiltechnik mbH, Aachen, Germany). Thereafter, graft polymerization of polyacrylic acid onto the plasma treated surface of the PVDF mesh samples was performed resulting in a polyacrylic acid monomer layer on the surface (PVDF + PAAc). The antibiotic gentamicin was bound to the active sites of the grafted mesh surface in three different concentrations respectively (PVDF + PAAc +2 μg/mg Gentamicin; PVDF + PAAc + 5 μg/mg Gentamicin, and PVDF + PAAc + 8 μg/mg Gentamicin). The efficacy of the antimicrobial mesh samples investigated by agar diffusion test, the gentamicin release from the mesh surface, and the cytotoxic side effects after direct contact to L929 mouse fibroblasts (BioWhittaker BE71-131F) were tested in a previously published study [20].

### Surgical procedure and observation periods

Operations were carried out under sterile surgical conditions and general anaesthesia by intramuscular administration of ketamine (Ketamin 10%, Sanofi-Ceva, Düsseldorf, Germany) and xylazine (Rompun 2%, Bayer, Leverkusen, Germany). Following the induction of anaesthesia, the skin was shaved and disinfected with polyvidone iodine solution (Braunosan Vet^®^, B. Braun Vet Care GmbH, Tuttlingen, Germany). Full thickness dermal incisions extending over 1.5 cm were performed 1 cm bilateral of the abdominal midline. Polymers (size: 0.5 × 0.5 cm) were implanted subcutaneously 1 cm distal of the xiphoid. In each animal two of the same polymeric mesh materials were implanted bilateral of the abdominal midline respectively. Following mesh implantation skin closure was obtained with 3/0 polypropylene (Prolene^®^, Ethicon Inc., Somerville, NJ, USA) single sutures. No additional antibiotic treatment was given before or during the experiments. Throughout the whole observation period all animals were objectively controlled and underwent daily clinical investigation to assess local and systemic complications. 7, 21 and 90 days after mesh implantation *n *= 5 animals in each group were euthanized by isoflurane (Attane™, MINRAD INC., Buffalo, NY, USA) asphyxation and decapitation. Tissue specimens for histological and immunohistochemical observations were immediately fixed in 10% formaldehyde.

### Histological assessment and immunohistochemical analysis

Briefly, all histological and immunohistochemical investigations including the cross polarization microscopy were performed in the same manner as previously described [17].

### Statistical analysis

Statistical analysis was carried out using the Statistical Package for Social Sciences (SPSS, Version 17.0, Chicago, IL, USA) software. Data were organized according to the types of meshes used (PVDF; PVDF + PAAc; PVDF + PAAc + 2 μg/mg; PVDF + PAAc + 5 μg/mg; PVDF + PAAc + 8 μg/mg), and to the duration of implantation (7, 21, 90 days). Analysis of histological and immunohistochemical parameters were performed using the Mann-Whitney *U *test. *P *values of < 0.05 were considered to be significant. All data are presented as mean ± standard deviation if not otherwise mentioned.

## Results

None of the animals died during the investigation and none of the animals developed any signs of local or general inflammation.

### MMP-2 protein expression

**Figure 1:**
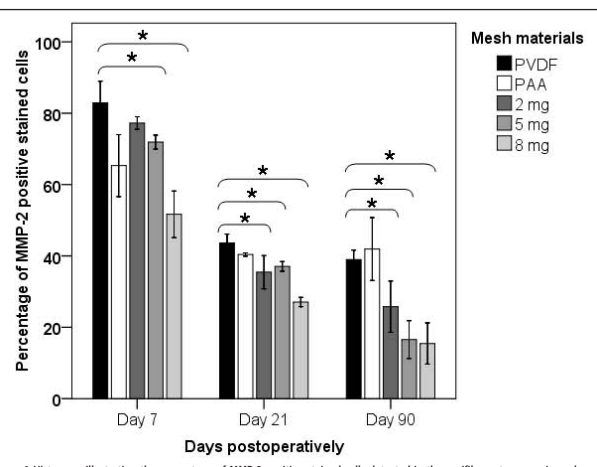
Histogram illustrating the percentage of MMP-2 positive stained cells detected in the perifilamentary area in each mesh group (represented as mean ± standard deviation, significant differences are marked (_*_)).

**Figure 2:**
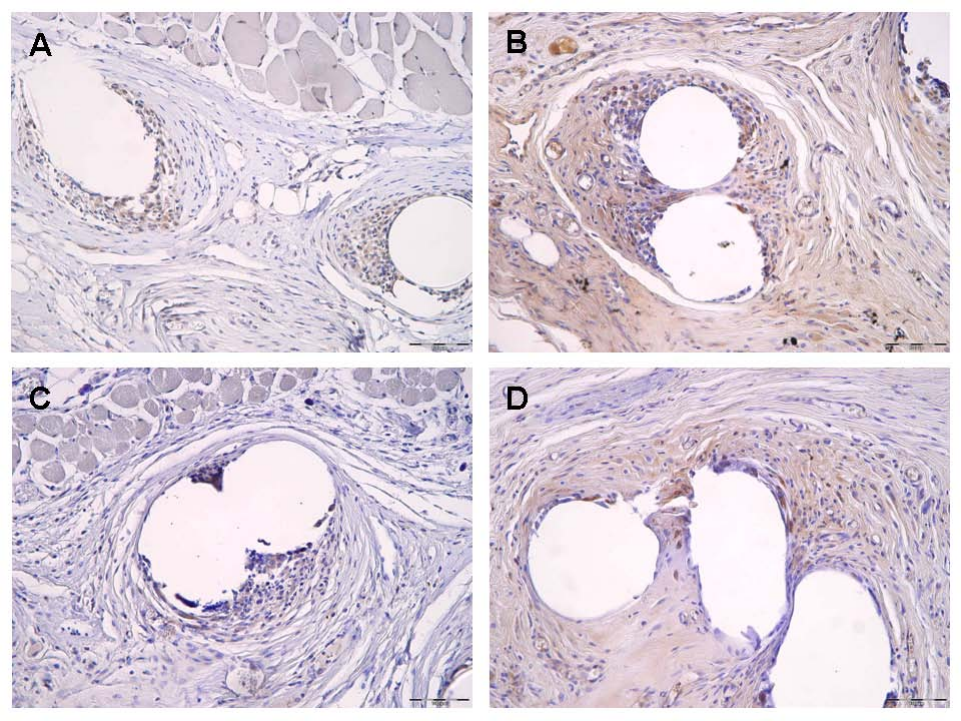
**Immunohistochemical images representing MMP-2 staining following implantation of a PVDF mesh (A) and a PVDF+PAAc+8 μg/mg mesh (C)**. Figure **(B)** demonstrates ß-galactosidase staining following implantation of a PVDF mesh and a PVDF+PAAc+8 μg/mg mesh **(D)** (magnification 200 fold, 90 days postoperatively respectively).

Apart from the PVDF + PAAc group, MMP-2 expression significantly decreased for all other mesh groups comparing day 7 to day 21 (PVDF: p = 0.016; 2 μg/mg: p = 0.016; 5 μg/mg: p = 0.016; 8 μg/mg: p = 0.029). In the later course of time the MMP-2 expression was significantly reduced only for the 5 and 8 μg/mg group comparing day 21 to day 90 (5 μg/mg: p = 0.016; 8 μg/mg: p = 0.029) (Figure 1 and 2A).

Both the 5 and 8 μg/mg mesh material showed significantly reduced levels of MMP-2 positive stained cells when compared to the pure PVDF group on day 7, 21 and 90 (5 μg/mg: p = 0.016, p = 0.016, p = 0.016; 8 μg/mg: p = 0.029, p = 0.016, p = 0.029). Except for day 7 the 2 μg/mg group likewise showed a significant reduction of MMP-2 protein expression compared to the pure PVDF group on day 21 (p = 0.016) and 90 (p = 0.032) (Figure 1 and 2A).

### MMP-2 promoter activity (ß-galactosidase)

**Figure 3:**
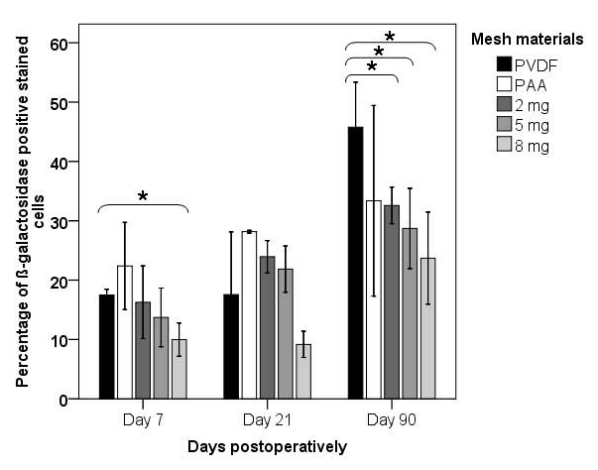
Histogram representing the perifilamentary ß-galactosidase expression in each mesh group (represented as mean ± standard deviation, significant differences are marked (_*_)).

To detect the perifilamentary MMP-2 promoter activity we analyzed the ß-galactosidase expression (Figure 2B). In contrast to the MMP-2 expression MMP-2 promoter activity was reciprocal and increased discrete over time for all mesh modifications. Certainly a significant increase of ß-galactosidase expression could be detected only for the 5 μg/mg group from day 7 to day 21 (5 μg/mg: p = 0.026). The enhancement of the MMP-2 promoter activity was significant for the PVDF (p = 0.016), 2 μg/mg (p = 0.008) and 8 μg/mg (p = 0.029) group comparing day 21 and day 90 (Figure 2B and 3).

On postoperative day 90 a significant reduction of ß-galactosidase stained cells was observed for all gentamicin supplemented mesh groups in comparison to the pure PVDF group (2 μg/mg: p = 0.016; 5 μg/mg: p = 0.016; 8 μg/mg: p = 0.029). On day 21 the MMP-2 promoter activity showed any significant differences comparing the groups. Only the 8 μg/mg group revealed a significant reduced expression of ß-galactosidase on day 7 compared to the pure PVDF group (p = 0.029) (Figure 2B and 3).

### Collagen type I/III ratio

**Figure 4:**
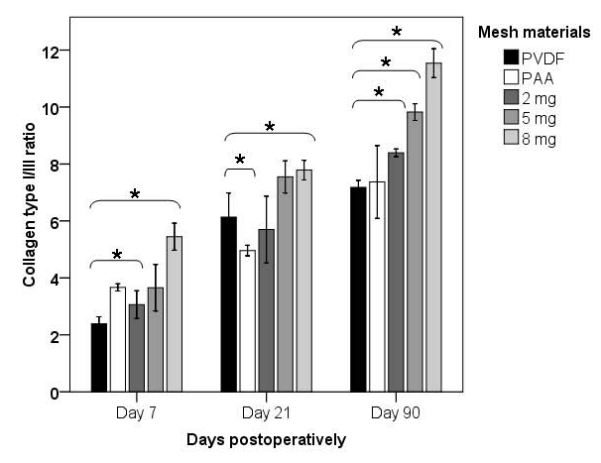
Histogram demonstrating the perifilamentary collagen type I/III ratio in each mesh group (represented as mean ± standard deviation, significant differences are marked (_*_)).

The collagen type I/III ratio was investigated to evaluate the quality of perifilamentary collagen deposition. This ratio ascended in each mesh group in the course of time. Comparing day 7 to day 21, the collagen type I/III ratio increased significantly in each mesh group except for the PVDF + PAAc group (PVDF: p = 0.016; 2 μg/mg: p = 0.016; 5 μg/mg: p = 0.016; 8 μg/mg: p = 0.029). A significantly increased collagen type I/III ratio was detected comparing day 21 to day 90 for each mesh group apart from the PVDF group (PVDF + PAAc: p = 0.036; 2 μg/mg: p = 0.008; 5 μg/mg: p = 0.016; 8 μg/mg: p = 0.029) (Figure 4).

Only the 8 μg/mg group showed significantly elevated values 7, 21 and 90 days after implantation when compared to the pure PVDF group (p = 0.029, p = 0.016, p = 0.029). The 2 μg/mg group as well as the 5 μg/mg group exhibited a significantly improved collagen type I/III ratio in comparison with the PVDF group only on day 90 (p = 0.016 and p = 0.016). Additionally the collagen type I/III ratio was significantly elevated in the 2 μg/mg group (p = 0.029) on day 7, and in the PVDF + PAAc group (p = 0.036) on day 21 compared to the PVDF group, respectively (Figure 4).

## Discussion

To reinforce the abdominal wall with mesh material is the gold standard in hernia repair and has lead to a considerable reduction of recurrence rates, however their implantation is associated with acute and chronic side effects like seroma and as long-term complication in particular chronic pain [13,14,21-24]. In particular long-term complications are supposed to be the consequence of a foreign body reaction (FBR), which is induced by every implantation of non-absorbable polymeric mesh material [25,26]. FBR offers characteristics of a chronic inflammatory reaction, which is marked by an activation of cytokine cascades and proteases, such as the matrix metalloproteinases 2 (MMP-2) [27,28]. This well known continuous expression of MMP-2 in foreign body reaction can also be observed in our study (Figure 1) whereas MMP-2 expression after injury is normally restricted to the proliferative phase of wound healing.

For this reason the inhibition of inflammation and as a consequence the normalization of MMP-2 expression is suggested as a new therapeutical approach to optimize mesh integration and to reduce mesh related complications [15,20]. By mesh modification with the aminoglycoside antibiotic gentamicin we found a time dependent reduction of the MMP-2 protein synthesis in a preceding study [17]. Especially, the perifilamentary MMP-2 protein expression was significantly diminished by mesh coating with a concentration of 8 μg/mg gentamicin at each time point. In previously published studies this potential impact of gentamicin on MMP-2 expression was likewise demonstrated, but in contrast to these results our current findings provide evidence for a presumably dose-dependent down-regulation of the MMP-2 [29,30]. Our current survey confirms the previously proven beneficial effect of gentamicin supplemented polyvinylidenfluoride (PVDF) mesh materials on tissue integration and foreign body reaction due to an improved collagen type I/III ratio and reduced MMP-2 protein expression.

However, and in contrast to our previous findings the MMP-2 promoter activity increased reciprocally to the MMP-2 protein expression, thereby suggesting that gentamicin represses the MMP-2 promoter in F8del mice. This ostensible controversy with an increased ß-galactosidase expression in F8del mice may be explained most suitable as follows. It is known that the extent of MMP-2 expression and enzymatic activity is regulated at the transcriptional, translational, and post-translational levels [31-33]. Specific regulatory elements governing MMP-2 gene transcription that reside up to -1686 base pairs (bps) relative of the translational start site have been identified [32,34-37]. A strong enhancer element named response element-1 (RE-1) is located at -1282/-1322 bps of the mice MMP-2 gene [32], which is evolutionarily conserved and is similarly operative within the human gene at -1657/-1619 bps relative to the transcriptional start site [38]. F8 mice harbor MMP-2 regulatory sequence -1686/+423, including the RE-1, showed both a reduced MMP-2 protein expression and promoter activity with presence of gentamcin [17]. On the other hand the F8del mice that were investigated in the present study harbour MMP-2 regulatory sequence -1241/+423, excluding the RE-1. This mouse strain revealed a reduced MMP-2 protein expression but a rising MMP-2 promoter activity in all mesh groups. Taken into account the preceding and current results, RE-1 mostly acts as a repressor of mesh induced MMP-2 transcription. Though we could confirm that gentamicin is beneficial for tissue integration regarding reduced MMP-2 expression and enhanced type I/III collagen ratio, the same repressive function of RE-1 could be detected for both the coated and the uncoated mesh group.

It is known that the aminoglycosides like gentamicin are able to penetrate eukaryotic cell membrane and it is known that beside its antibacterial activity gentamicin interacts with intracellular molecules thereby influencing intracellular pathways, which might explain the reduced MMP-2 expression and gene regulation [39,40]. Although the exact molecular mechanisms are not yet unraveled, our findings support the hypothesis that the observed down-regulation of MMP-2 is finally mediated by inhibition of the MMP-2 promoter.

Certainly it remains unclear which transcription factors mediate the down-regulation of mesh induced MMP-2 expression. At least distinct transcription factors have been shown to bind to the RE-1. These include *activating protein-2 *(AP 2) [37], *Y-box protein-1 *(YB-1) [38], *signal transduction and activator of transcription factor 3 *(Stat3) [41], and *p53 *[36]. In a previously published study by *Jansen *et al. it could be shown that macrophages that are adjacent to the mesh filaments play a crucial role for mesh induced MMP-2 expression [9]. Therefore, the gentamicin mediated down-regulation of MMP-2 transcription in our study might be due to a cellular effect, e.g. by reducing infiltration or activation of macrophages or other inflammatory cells that are involved in MMP-2 expression. The results of our current investigation further support the hypothesis of an intercellular crosstalk between inflammatory cell like macrophages and fibroblasts via MMP-2 regulation in the process of foreign body reaction.

Restrictively, it has to be noted that the collagen type I/III ratio was significantly elevated for each gentamicin concentration on day 90, whereas on postoperative day 7 and 21 merely the mesh with a coating of 8 μg/mg gentamicin constantly resulted in a significant improvement of the collagen ratio. As already mentioned a dose-dependent effect of gentamicin is possibly the reason for an insignificant or even negative effect on collagen formation and degradation. This is in line with a study by *Asch *and *Farnham *who demonstrated a dose-dependent effect of gentamicin on the protease activity of collagenase derived from Clostridium histolyticum [42].

## Conclusion

The present results again demonstrate a beneficial effect of gentamicin on chronic foreign body reaction by modulation of MMP-2 gene transcription that may be a feasible approach to optimize mesh integration into the abdominal wall, and ultimately to improve the long-term outcome following hernia mesh repair. However, animal models have their natural limitations, and results cannot be extrapolated directly to the situation in humans. Therefore, clinical trials are needed to verify the suggested beneficial effect of gentamicin, and further experimental studies are needed to elucidate the exact molecular mechanisms of gentamicin on MMP-2 gene transcription.

## Competing interests

The authors declare that they have no competing interests.

## Authors' contributions

**MB **carried out the animal experiments and immunohistochemical investigations, was involved in acquisition, analysis and interpretation of the data, drafted and revised the manuscript and gave the final approval of the version to be published. **KTvT **carried out the animal experiments, was involved in acquisition of the data and was involved in the statistical analysis, participated in drafting and revising the manuscript and gave his final approval of the version to be published. **CR **carried out the immunohistochemical investigations, participated in drafting the manuscript and gave her final approval of the version to be published. **CDK **was involved in acquisition and interpretation of the data, participated in drafting and revising the manuscript and gave his final approval of the version to be published. **KJ **made substantial contributions in analysis and interpretation of the data was involved in drafting and revising the manuscript and gave his final approval of the version to be published. **JC **made substantial contributions in analysis and interpretation of the data was involved in drafting and revising the manuscript and gave his final approval of the version to be published. **MJ **was a major contributor in planning and designing the study, helped in drafting the manuscript and gave his final approval of the version to be published. **UPN **made substantial contributions in interpretation of the data was involved in drafting and revising the manuscript and gave his final approval of the version to be published. **PLJ **conceived the study, participated in its design and coordination and helped in drafting and revising the manuscript and gave her final approval of the version to be published.

## Pre-publication history

The pre-publication history for this paper can be accessed here:

http://www.biomedcentral.com/1471-2482/12/1/prepub
